# Real-world effectiveness and safety of fingolimod in patients with multiple sclerosis in the Czech Republic: results from core and extension parts of the GOLEMS study up to 48 months

**DOI:** 10.1186/s12883-022-02656-8

**Published:** 2022-04-15

**Authors:** Veronika Tichá, Zuzana Počíková, Josef Vytlačil, Radka Štěpánová

**Affiliations:** 1 MS Center, Department of Neurology and Center of Clinical Neuroscience, First Faculty of Medicine and General University Hospital in Prague, Charles University, Katerinska 30, 12000 Praha 2, Prague, Czech Republic; 2 Novartis s.r.o., Prague, Czech Republic; 3 ANOVA CRO s.r.o., Zelená 2002/21a, 160 00 Prague 6 – Dejvice, Prague, Czech Republic

**Keywords:** Fingolimod, Multiple sclerosis, GOLEMS extension study, Relapse, EDSS score

## Abstract

**Background:**

Fingolimod, an oral sphingosine 1-phosphate receptor immunomodulator, is approved in Europe for people with multiple sclerosis (pwMS) with highly active disease despite a full and adequate course of treatment with ≥ 1 disease-modifying therapy or patients with rapidly evolving severe relapsing–remitting MS. GOLEMS, a national, multicenter, non-interventional, single-arm, real-world study showed a favorable benefit–risk profile of 12-month treatment with fingolimod in pwMS in the Czech Republic. Here, we evaluated the long-term effectiveness and safety of fingolimod and its impact on disability progression and work capability for up to 48 months in pwMS.

**Methods:**

The endpoints assessed were the incidence and severity of MS relapses in fingolimod-treated patients and the proportion of relapse-free patients up to 48 months of fingolimod treatment, change from baseline in the Expanded Disability Status Scale (EDSS) score, and change from baseline in work capability assessment. Efficacy outcomes were analyzed in the completed and efficacy sets, and safety was evaluated in all the enrolled patients.

**Results:**

Of 240 enrolled patients, 237 were included into efficacy set. Patients with a minimum of a 12-month observation period in the core study who continued fingolimod treatment, were eligible to participate in the extension phase. Of 211 patients enrolled in extension study, 155 were evaluated in the completed set. Based on analysis of 48-month period of fingolimod treatment, 95/237 patients (40.1%) in the efficacy set, 54/155 (34.8%) in the completed set were free of relapses. The majority of relapses reported were moderate in intensity. Mean EDSS score remained stable throughout 48-month study period (Baseline, 3.4; Month 48, 3.6). No trend was observed in changes in work capability assessment or number of missed days of work. Of 240 enrolled patients, 147 (61.3%) had ≥ 1 treatment-emergent adverse event (AE) and 20 (8.3%) reported serious AEs. In total, 45 patients (18.8%) permanently discontinued treatment because of AEs related to study drug; two patients reported pregnancy after treatment initiation and subsequently discontinued the treatment; no deaths were reported.

**Conclusion:**

GOLEMS study demonstrated the sustained effectiveness and manageable safety profile of fingolimod under real-world conditions over 48 months in patients with MS.

**Trial registration:**

Not applicable.

## Background

Multiple sclerosis (MS) is a chronic, autoimmune, inflammatory, demyelinating disease of the central nervous system (CNS) [1, 2] affecting approximately 2.3 million individuals worldwide [3]. Patients with MS require long-term treatment with disease-modifying therapies that can reduce the number of relapses and delay the progression of disability [4, 5].

Fingolimod (FTY720, Gilenya®; Novartis Pharma AG) is a first-in-class, oral sphingosine 1-phosphate (S1P) receptor immunomodulator that acts as a functional antagonist by internalizing activated receptors [1]. S1P down-regulation prevents lymphocyte egress from lymphoid tissues, thereby reducing autoaggressive lymphocyte infiltration into the CNS. In Europe, fingolimod is approved for people with MS (pwMS) with highly active disease despite a full and adequate course of treatment with at least one disease-modifying therapy or patients with rapidly evolving severe relapsing–remitting MS (RRMS) [6]. As of 31 May, 2020, it is estimated that > 303,600 patients have been treated with fingolimod, corresponding to > 808,900 patient-years of exposure in clinical trials and the post-marketing setting (Novartis Data on file)**.**

The three large Phase 3 clinical trials of fingolimod—FREEDOMS [7], FREEDOMS II [8], and TRANSFORMS [9]—showed a significant reduction in relapse rate, magnetic resonance imaging-related lesion counts, disability progression, and brain volume loss versus placebo and intramuscular interferon β-1a in patients with RRMS. These effects were sustained in the respective extension studies [10, 11], which demonstrated decreased MS disease activity and disability progression. In addition, the long-term observational study (LONGTERMS) also confirmed the safety and efficacy associated with the fingolimod treatment for up to 14 years in patients with relapsing MS [12]. Real-world evidence studies complement the randomized controlled trials by contributing to the understanding of long-term effectiveness and tolerability of therapies to uncover the best treatment paths in routine clinical practice [13].

The Gilenya (FingOLimod) in prescribing conditions defined by the CzEch regulator of drug reiMburSement (GOLEMS) study was planned to provide the requested healthcare outcomes data from pwMS receiving fingolimod under real-life conditions to the Health Authority and General Health Insurance Company of the Czech Republic [14]. The results from the 12-month GOLEMS study showed a favorable benefit–risk profile of fingolimod consistent with the pivotal Phase 3 trials [14].

Here, we report the results from the core and extension parts of GOLEMS study, which evaluated the long-term effectiveness and safety of fingolimod treatment for up to 48 months in patients with MS.

## Materials and methods

### Study design

GOLEMS was a national, multicenter, observational, non-interventional, single-arm study in the Czech Republic, conducted between November 2012 and March 2018 [14]. All existing MS centers in the Czech Republic were asked to participate in this study. As this was a non-interventional study, there were no special protocol-mandated visits or procedures associated with the study. Overall, in the core and extension, patients in the study were observed for a period of 4 years. Data recorded during observations at the second, third, and fourth year of the study were used for the evaluation of long-term therapy outcomes. All patients were assessed and monitored as per the revised label issued on March 20, 2012. Fingolimod prescription was at the discretion of both the treating physician and the patient and was independent of participation in the study.

### Patient population

Patients with a minimum of a 12-month observation period in the core study [14], who continued treatment with fingolimod, and had signed an informed consent form were eligible to participate in the extension phase. In total, 240 patients were enrolled in the core GOLEMS study and 211 patients entered the extension study.

The study was conducted in compliance with the ethical principles of the Declaration of Helsinki and the International Conference on Harmonization Good Clinical Practice Guidelines [15]. The protocol was approved by an independent ethics committee or institutional review board for each study site/hospital. All patients or their legal representatives provided written informed consent before commencing the trial-related procedures and allowed the results to be part of a publication.

### Study endpoints

The endpoints assessed were incidence and severity of MS relapses during the whole 48-month treatment period; change from baseline in Expanded Disability Status Scale (EDSS) score at each post-baseline visit; and changes in work capability assessment (Work Productivity and Activity Impairment Questionnaire-General Health [WPAI-GH] scores and number of missed days of work) from baseline to the 48 months.

The proportion of relapse-free patients and time to first relapse were also analyzed for subgroups of patients with EDSS scores of ≤ 3 and > 3 at baseline. In addition, the proportion of relapse-free patients in a subgroup of patients treated with natalizumab before fingolimod initiation and the factors influencing the occurrence of an MS relapse were also assessed. Safety was assessed by adverse events (AEs), serious AEs (SAEs), performance of first-dose observation, ophthalmic examinations, and laboratory assessments (liver function tests and lymphocyte counts).

Medical health record data were collected by the investigators using the web-based software OpenClinica®, an electronic data capture system compliant with the guidelines of Good Clinical Practice (21 CFR Part 11) [14].

### Statistical analyses

As the study was non-interventional and objectives were exploratory, no formal hypothesis was set for this study. For categorical data, counts and percentages were reported; for efficacy variables, 95% confidence intervals (CIs) for percentages were calculated. For continuous data, descriptive statistics including number of available observations, mean, standard deviation, median, lower and upper quartiles, minimum and maximum were reported; for efficacy variables, 95% CIs for the mean or median were calculated. Time to first MS relapse after treatment start was presented using Kaplan–Meier curves. For evaluation of factors which might influence an occurrence of an MS relapse, the logistic regression was used. Odds ratio together with 95% Wald CI and its *p*-value were reported. All statistical tests were performed on exploratory basis only.

In this study, the outcomes were assessed in two different sets of patient populations. The efficacy set (*N* = 237) comprised all treated patients except for three who discontinued the study treatment at Month 3. The reason for discontinuation was neither due to efficacy nor safety issues. The completed set (*N* = 155) included all patients who completed the 48-month treatment period (even if the patient temporarily discontinued the study).

Efficacy data were evaluated in the efficacy and completed sets. The primary analysis was performed using the completed set. The safety analysis was performed in the safety set, consisting of all enrolled patients.

## Results

### Patient disposition and demographics

A total of 240 patients were enrolled in the study. Out of 240 patients at the start of the study, 211 patients entered the extension and 155 completed the 48-month period. Of 240 enrolled patients, 65 patients permanently, 20 patients temporarily discontinued the treatment (e.g. due to pregnancy, AEs), and 29 patients did not continue in the study extension. The reasons for discontinuation of treatment in patients in whom this information was available were unsatisfied treatment effect (*n* = 20), AEs (*n* = 20), illness progression (*n* = 8), patient’s request (*n* = 8), administrative problems (*n* = 2) and patient lost to follow-up (*n* = 1). Of the overall patient population in the GOLEMS core study, 70.4% of patients (169/240) were women, the average age was 37 years at baseline, and the mean occurrence of the first symptoms of MS was 10.35 years before the study entry. During the year prior to fingolimod treatment initiation, 19.6% of patients had no relapse, 22.5% had one relapse, 57.9% had two or more relapses. The maximum number of relapses in one patient within the year prior to fingolimod treatment was four. During the 2 years prior to fingolimod treatment, 45% of patients had > 2 relapses. Mean EDSS score at baseline was 3.4 and the mean number of missed days of work during the 3 months prior to study entry was 7.9 days (*n* = 24) (Table 1).

**Table 1:** Patient demographics and baseline characteristics

Parameter	*N* = 240
Age, years	
Mean (± SD)	37.4 (± 9.27)
Median (Q_1_; Q_3_)	37.0 (31.0; 43.0)
Min; Max	18; 67
Sex, n (%)	
Male	71 (29.6)
Female	169 (70.4)
Time since first MS symptom prior to study entry, years	
Mean (± SD)	10.35 (± 6.70)
Median (Q_1_; Q_3_)	9.42 (5.22; 14.26)
Min; Max	0.4; 38.5
Frequency of relapses during the year prior to fingolimod treatment, n (%) [95% CI]	
Patients with no relapse	47 (19.6) [14.8; 25.2]
Patients with 1 relapse	54 (22.5) [17.4; 28.3]
Patients with 2 relapses	102 (42.5) [36.2; 49.0]
Patients with > 2 relapses	37 (15.4) [11.1; 20.6]
Number of MS relapses within the year prior to fingolimod treatment	
Mean (± SD)	1.6 (± 1.02)
Median (Q_1_; Q_3_)	2.0 (1.0; 2.0)
Min; Max	0; 4
Frequency of relapses in the 2 years prior to fingolimod treatment, n (%) [95% CI]	
Patients with no relapse	35 (14.6%) [10.4; 19.7]
Patients with 1 relapse	27 (11.3) [7.6; 15.9]
Patients with 2 relapses	70 (29.2) [23.5; 35.4]
Patients with > 2 relapses	108 (45.0) [38.6; 51.5]
Number of MS relapses in the 2 years prior to fingolimod treatment	
Mean (± SD)	2.3 (± 1.46)
Median (Q_1_; Q_3_)	2.0 (1.0; 3.0)
Min; Max	0; 9
EDSS score for the 3 months prior to study entry or at the start of fingolimod treatment	
Mean (± SD)	3.4 (± 1.26)
Median (Q_1_; Q_3_)	3.5 (2.5; 4.5)
Min; Max	0; 6
Number of missed days of work during the 3 months prior to study entry	
Mean (± SD)	7.9 (± 19.66)
Median (Q_1_; Q_3_)	0.0 (0.0; 6.0)
Min; Max	0.0; 90.0

*CI* Confidence interval, *EDSS* Expanded Disability Status Scale, *Max* Maximum, *Min* Minimum, *N* Number of patients, *Q*_*1*_ Lower quartile, *Q*_*3*_ Upper quartile, *SD* Standard deviation

### Incidence and severity of MS relapses in patients treated with fingolimod for 48 months

**Fig. 1:**
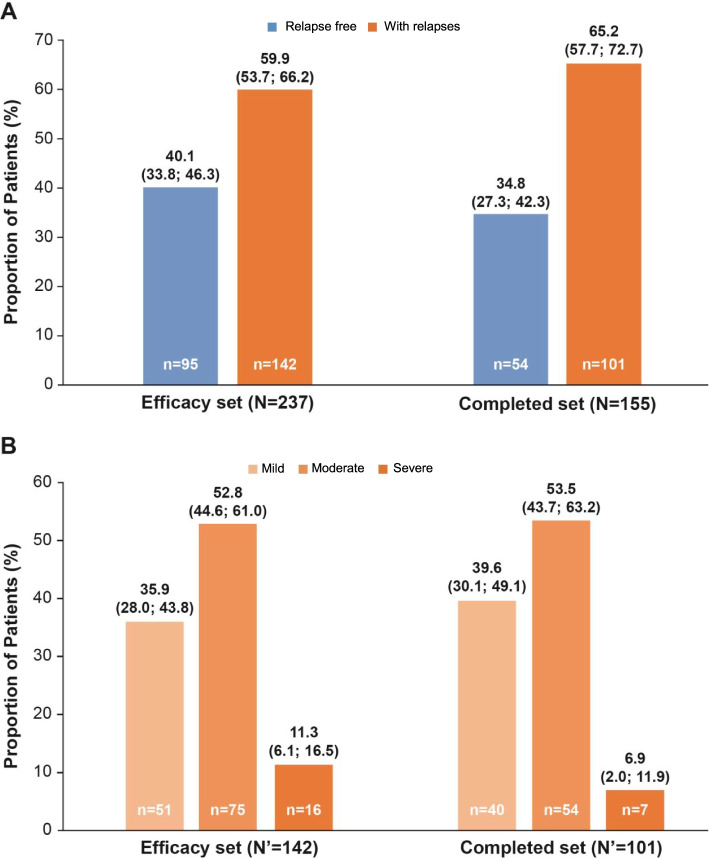
Proportion of patients with and without relapses (**A**) and severity of relapses during 48 months of treatment with fingolimod (**B**). Data presented as percentage of patients (Wald 95% CI) CI, confidence interval; MS, multiple sclerosis; N, total number of patients in the set; N’, total number of patients with relapses; n, number of patients with the outcome

Overall, 40.1% in the efficacy set and 34.8% in the completed set were free of relapses (Fig. 1A). In patients who experienced relapses, the majority of relapses reported were moderate in intensity (efficacy set [52.8%] and completed set [53.5%]) (Fig. 1B).

**Fig. 2:**
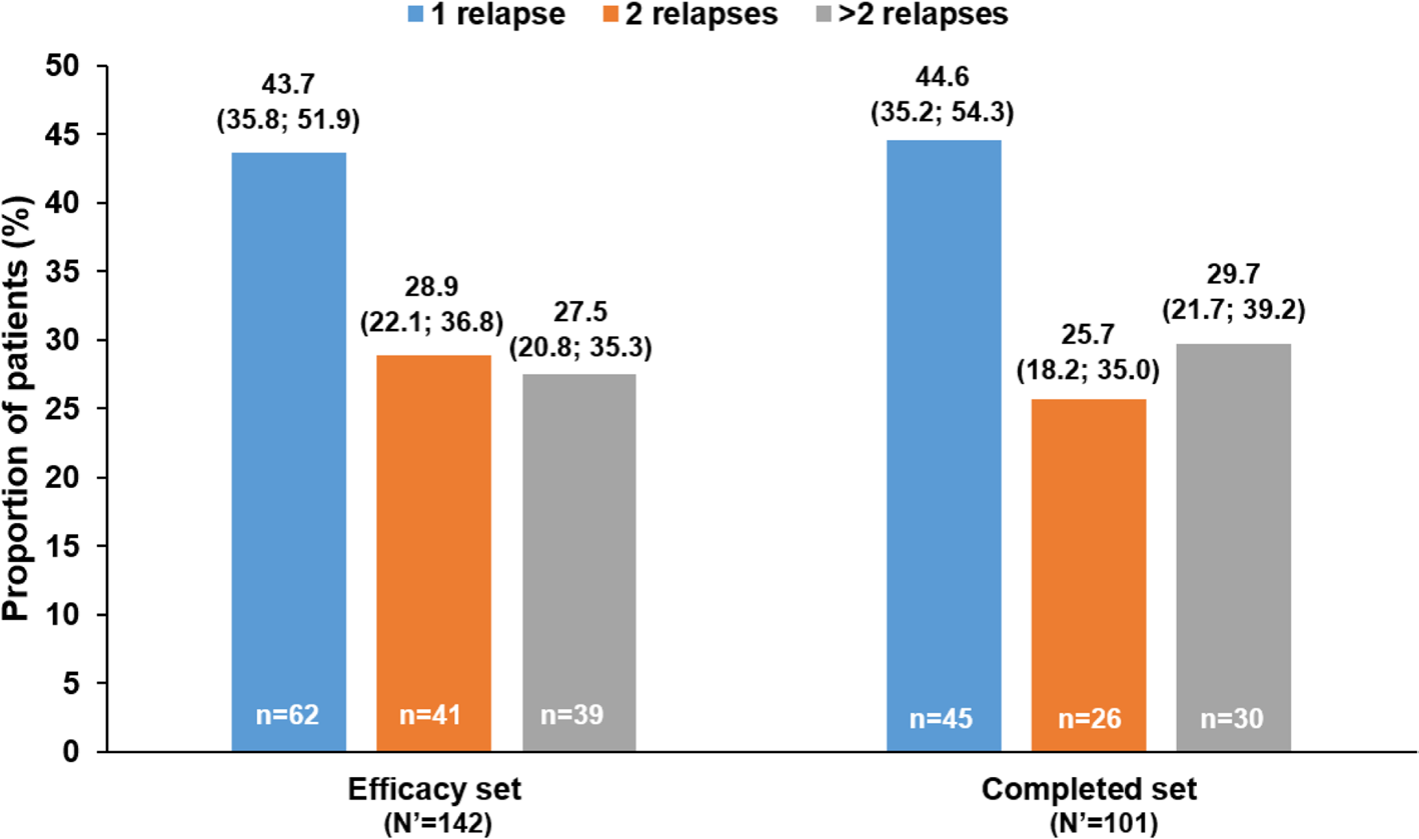
Frequency of MS relapses during 48 months of treatment with fingolimod. Data presented as percentage of patients (Wald 95% CI) CI, confidence interval; MS, multiple sclerosis; N’, total number of patients with relapses; n, number of patients with the individual outcomes

Among patients with relapses, 43.7% (62/142) in the efficacy set and 44.6% (45/101) in the completed set had one relapse during 48 months (Fig. 2). The mean number of relapses per patient per year in the efficacy set was 0.55 (95% CI: 0.45; 0.66) and in the completed set was 0.36 (95% CI: 0.30; 0.43).

**Fig. 3:**
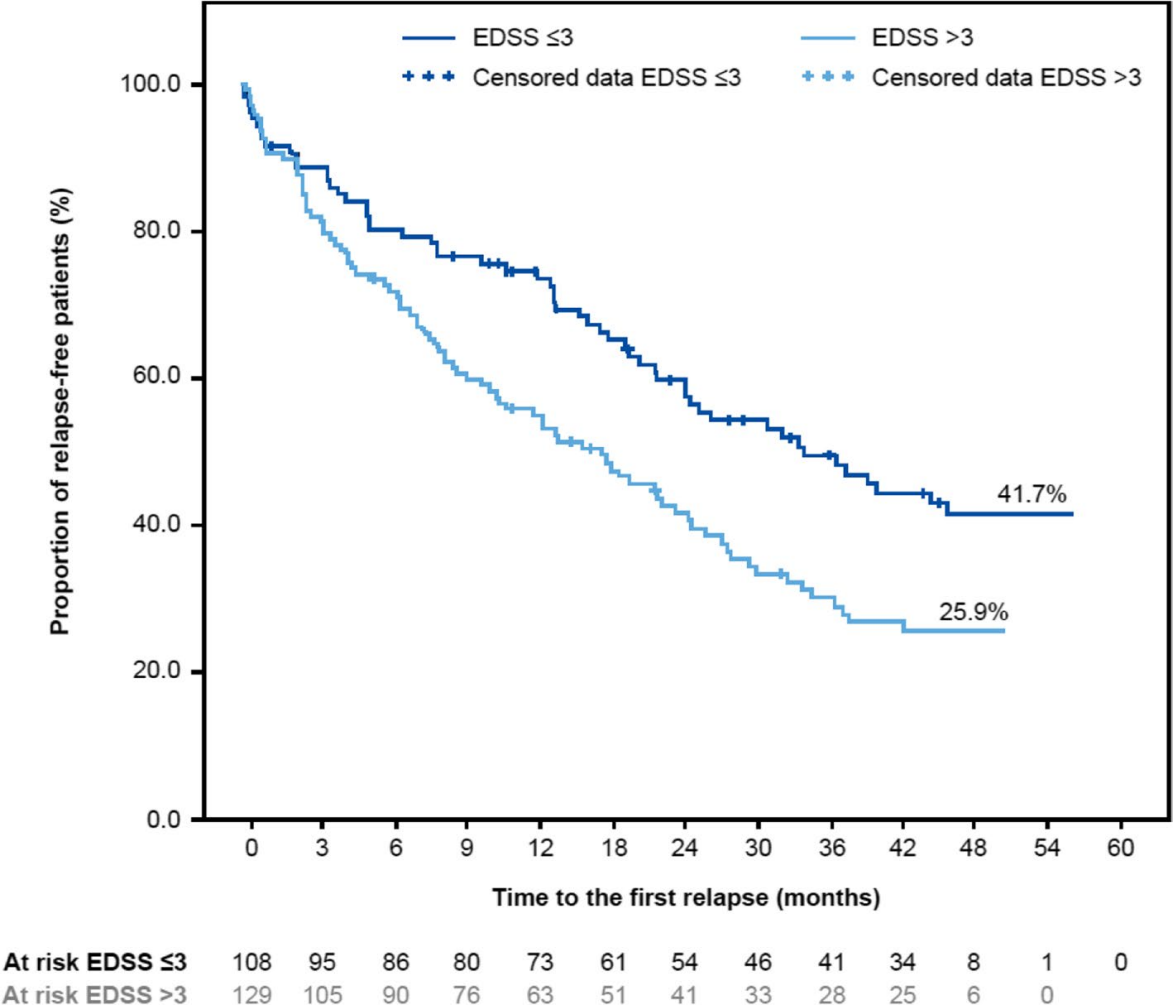
Time to first MS relapse after the start of fingolimod treatment (efficacy set, by EDSS score at baseline) EDSS, Expanded Disability Status Scale; MS, multiple sclerosis

The proportion of relapse-free patients was higher in the subgroup of patients with a baseline EDSS score ≤ 3 versus those with an EDSS score > 3 (Fig. 3). In the efficacy set, 41.7% of patients with a baseline EDSS score ≤ 3 were relapse-free versus 25.9% with a baseline EDSS score > 3. Similarly, in the completed set, 36.7% with a baseline EDSS score ≤ 3 were relapse-free versus 28.9% with an EDSS score > 3.

### Distribution of severity of relapses over time

The proportion of relapse-free patients decreased evenly over time. In the efficacy set, 91.1% of patients were relapse free at Month 1 and 40.1% were relapse free at Month 48. Similarly, in the completed set, 92.9% were free from relapses at Month 1 and 34.8% were relapse free at the Month 48 visit.

**Fig. 4:**
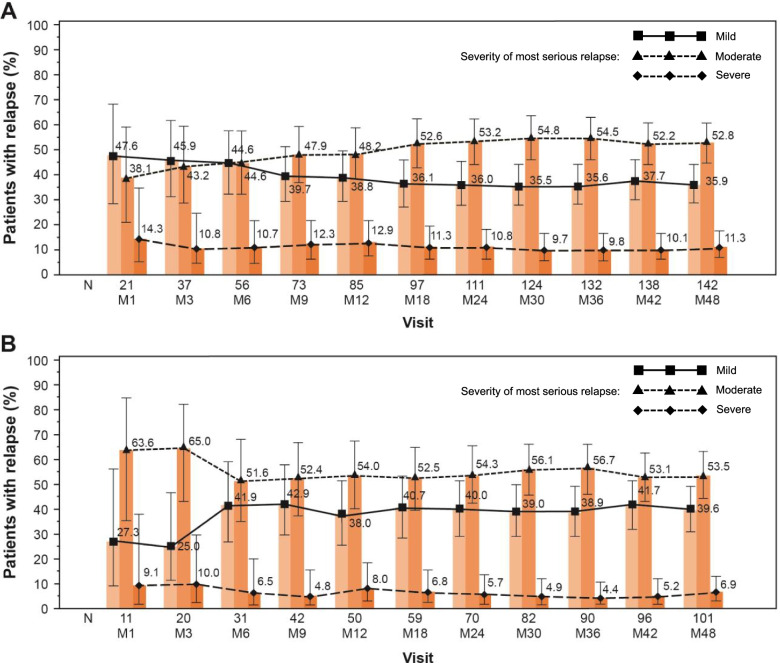
Severity of MS relapses that occurred in the efficacy set (A) and completed set (B), by visit Data presented as percentage of patients (Wilson 95% CI) CI, confidence interval; M, Month; MS, multiple sclerosis

In the efficacy set, the number of relapses reported as mild decreased and the number of patients with a moderate relapse slightly increased; in the completed set, moderate relapses were more frequent than mild relapses from the beginning to the end of treatment. The number of severe relapses remained stable in both of these sets (Fig. 4).

### Factors influencing the occurrence of an MS relapse

In the efficacy set, age, EDSS score, and number of relapses during the 2 years prior to treatment significantly correlated with the occurrence of MS relapse in the first 48 months of fingolimod treatment (Table 2). With every 10-year increase in the age at baseline, the chance of being free of relapses was almost 1.5 times higher. For every 1-point increase in baseline EDSS score, the chance of being free of relapses was about 20% lower. If the number of relapses during the last 2 years was higher by one relapse, then the chance of being relapse free was about 30% lower. In the completed set, none of the variables in the model showed a significant effect on being relapse free.

**Table 2:** Factors influencing the occurrence of an MS relapse for the first 48 months of fingolimod treatment (efficacy set, *N* = 237)

Parameter	Unit	*p*-value	Odds ratio (Wald 95% CI) estimate for being "relapse free"
**Age at baseline, years**	**10.0**	**0.0372**	**1.467 (1.023; 2.104)**
**EDSS score at baseline**	**1.0**	**0.0335**	**0.765 (0.598; 0.979)**
Number of relapses during the year before the start of fingolimod treatment	1.0	0.7551	1.088 (0.641; 1.845)
**Number of relapses during the 2 years before the start of fingolimod treatment**	**1.0**	**0.0452**	**0.680 (0.467; 0.992)**
Severity of the last relapse before the start of fingolimod treatment	1.0	0.4669	0.829 (0.500; 1.374)
Disease duration (days)	1.0	0.1720	1.000 (1.000; 1.000)
Type of previous MS therapy^a^			
Group 2 vs Group 1	1.0	0.5451	0.787 (0.337; 1.835)
Group 3 vs Group 1	1.0	0.6987	1.015 (0.506; 2.035)

Rows indicated in bold format show statistically significant data. Duration of the diagnosis was calculated as the difference between the date of starting fingolimod treatment and the date of diagnosis

*CI* Confidence interval, *EDSS* Expanded Disability Status Scale, *MS* Multiple sclerosis, *N* Number of patients

^a^Patients were categorized into three groups according to the type of the previous therapy of MS: Group 1 – patients treated with any interferon or glatiramer acetate; Group 2 – patients treated with natalizumab in a period of at least 3 months before initiating fingolimod treatment; Group 3 – patients treated with another medication in addition to the medications stated above

### EDSS scores and work capability assessment

**Fig. 5:**
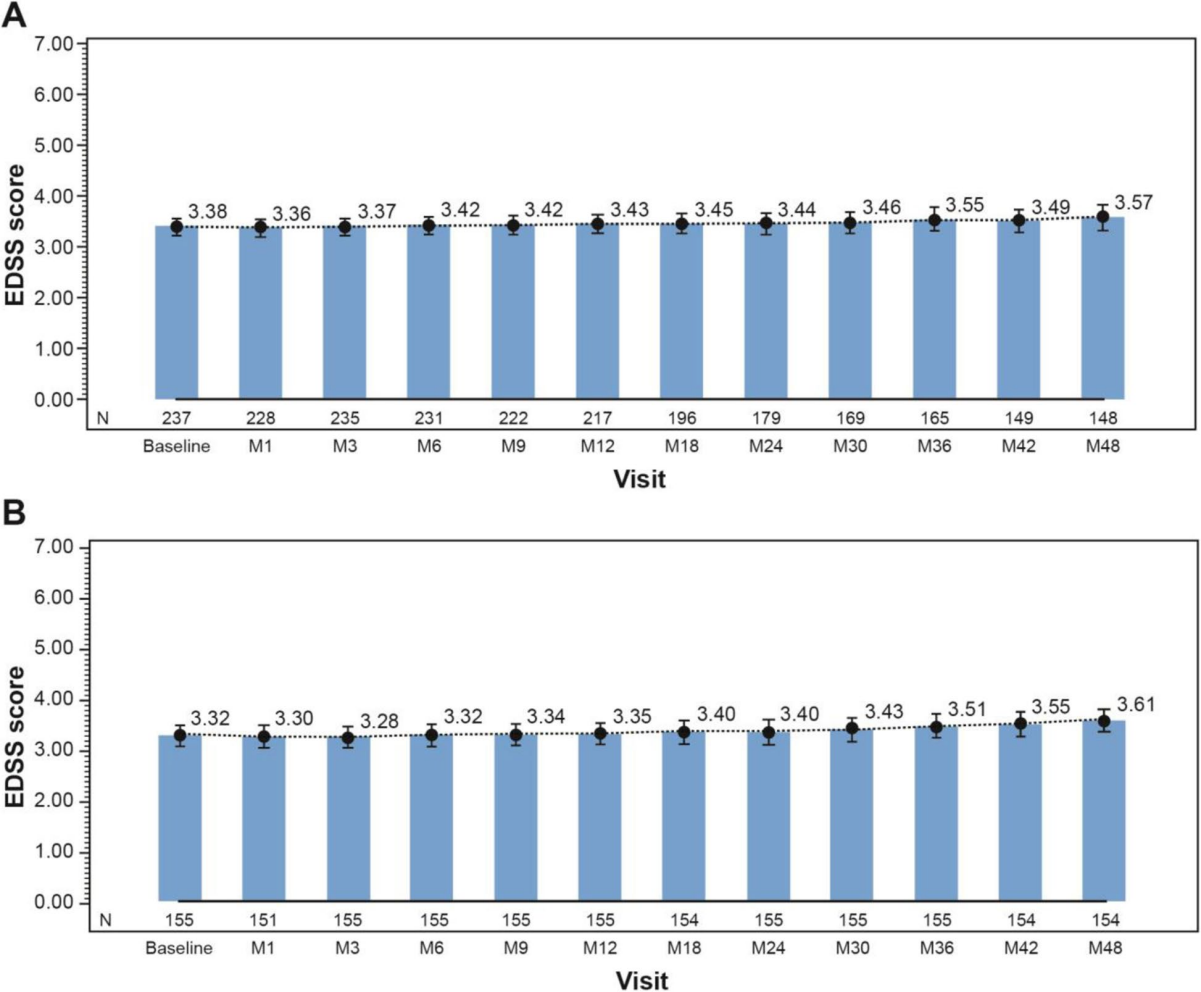
EDSS scores in the efficacy set (**A**) and the completed set (**B**), by visit Data presented are Mean (95% CI) CI, confidence interval; EDSS, Expanded Disability Status Scale; M, Month, N, number of patients

EDSS scores were found to be stable over the observation period and no significant changes were observed during fingolimod treatment in both the efficacy and completed sets (Fig. 5). Median EDSS score was around 3.5 at each visit and around 4.5 at the time of relapses.

Analysis of the WPAI-GH questionnaire, number of missed days of work, changes in work capability assessment, and changes in the number of missed days of work did not reveal any trends.

### Relapse status in patients receiving natalizumab before fingolimod initiation

At Month 48, among 59 patients who had received natalizumab one year before fingolimod treatment initiation, 19 (32.2%) were relapse free after 48 months of treatment. Of 40 patients with relapses, 16 (40%) had mild relapses, 18 (45%) had moderate relapses, and 6 (15%) had severe relapses.

### Safety results

Overall, 147 (61.3%) patients from safety set reported 222 AEs (Table 3). Among these, 100 AEs were related to the study drug in 76 patients (31.7%), and 21 AEs in 20 patients (8.3%) were reported as SAEs (Table 4).

**Table 3:** Summary of AEs (Safety set) (*N* = 240)

Parameter	AE count	n (%)
**Any treatment-emergent AE**	222	147 (61.3)
**Relationship to study drug**		
No	122	89 (37.1)
Yes	100	76 (31.7)
**Outcome**		
Completely recovered	140	94 (39.2)
Condition still present and unchanged	46	42 (17.5)
Condition improving	27	26 (10.8)
Recovered with sequelae	6	6 (2.5)
Condition deteriorated	2	2 (0.8)
Completely recovered, condition still present and unchanged	1	1 (0.4)

*AE* Adverse event, *n* Number of patients, *N* Total number of patients

**Table 4:** Summary of SAEs (Safety set) (*N* = 240)

Parameter	SAE count	n (%)
**Any treatment-emergent SAE**	21	20 (8.3)
**Relationship to study drug**		
No	12	11 (4.6)
Yes	9	9 (3.8)
**Outcome**		
Completely recovered	10	9 (3.8)
Condition still present and unchanged	3	3 (1.3)
Condition improving	3	3 (1.3)
Recovered with sequelae	4	4 (1.7)
Completely recovered, condition still present and unchanged	1	1 (0.4)
**SAEs**		
MS relapse	4	4 (1.7)
Hepatopathy	2	2 (0.8)
Epileptic seizure	2	1 (0.4)
Breast cancer	1	1 (0.4)
Bronchopneumonia	1	1 (0.4)
Cerebral infarction	1	1 (0.4)
Cerebrovascular accident	1	1 (0.4)
Disease progression	1	1 (0.4)
Gynecological infection	1	1 (0.4)
Herpes zoster	1	1 (0.4)
Humerus fracture	1	1 (0.4)
Insufficient treatment effect	1	1 (0.4)
Joint and muscle pain	1	1 (0.4)
Macular edema	1	1 (0.4)
Rebound of MS	1	1 (0.4)
Spastic bronchitis	1	1 (0.4)

*n* Number of patients, *N* Total number of patients, *SAE* Serious adverse event

Overall, 45 patients (18.8%) experienced AEs leading to permanent discontinuation of fingolimod treatment. There were no deaths observed in this study. The liver function tests were abnormal at baseline in 4.6% of patients and in 15.3–29.4% of patients during treatment. The mean lymphocyte count ranged from 0.571 × 10^9^/L to 0.654 × 10^9^/L during the 12 months of fingolimod treatment. The ophthalmologic examination was abnormal for one patient at the baseline, Month 9, and Month 12 visits but there are large proportions of missing data for this examination.

## Discussion

The GOLEMS study evaluated the long-term efficacy and safety outcomes of fingolimod treatment for up to 48 months in patients with MS. In this study, 40.1% of patients treated with fingolimod were free of relapses over a period of 48 months. The number of relapse-free patients decreased evenly over time. EDSS score did not change significantly over time and no significant impairment was recorded. Changes in work capability assessment analyzed by the WPAI-GH questionnaire and the number of missed days of work did not reveal any trends. Out of 240 patients, 147 had at least one treatment-emergent AE and only 20 (8.3%) patients had SAEs. Age, EDSS score at baseline, and number of relapses during the 2 years prior to fingolimod treatment significantly correlated with the occurrence of MS relapse during the 48 months of fingolimod treatment.

Similar to GOLEMS, PANGAEA—a prospective, observational, 5-year registry study—was another real-world study that determined the incidence of safety-related parameters and monitored the general safety profile of fingolimod in routine practice in Germany. The GOLEMS study had similar results to the 36-month interim results of the PANGAEA study (*n* = 1518) [16]. At 36 months of PANGAEA study, more than 58.2% of patients were free from relapses. At 48 months of GOLEMS study, 40.1% were relapse-free indicating that the percentage of relapse-free patients decrease over time.

After 48 months of fingolimod treatment in GOLEMS, the average number of relapses per patient per year was 0.36 in the completed analysis set compared with 0.29 in the interim analysis of PANGAEA (*N* = 4229) [17]. In the GOLEMS study, the mean number of relapses per patient per year in the 12 months before fingolimod initiation was 1.6, which was comparable to the 1.5 relapses in PANGAEA, with a relative reduction of 67% and 72%, respectively.

During the 36-month PANGAEA study, EDSS scores remained stable and only a small proportion of patients (< 10%) experienced confirmed disability worsening during any 12-month follow-up period. In addition, improvements in EDSS scores were observed in an increasing proportion of patients in each 12-month follow-up period [16]. Similarly, patients in the GOLEMS trial treated with fingolimod did not show any significant changes in EDSS score during the treatment period.

The types of AEs were similar in both studies and results did not show any new safety signals related to fingolimod treatment.

As there were no specific protocol-mandated visits in the study, evaluations were determined by treating physicians according to the local prescribing information. However, the non-interventional design of the study reflects real-life data.

One of the major limitations of this study was the large amount of missing data for the assessment of changes in work capability due to the low response rate (~ 10%) in completing the WPAI-GH questionnaires, which prevented any meaningful statistical interpretation. Another limitation of the study include the number of discontinuations throughout the study; of 240 enrolled patients, 155 (65%) completed the 48-month study period. Lack of MRI data was another limitation because the trial was conducted in MS centers which used different MRI machines and software. It was not possible to set up a unified standard protocol for quantitative analysis of MRI parameters.

## Conclusion

Long-term treatment with fingolimod showed sustained efficacy on disease activity measured by presence and severity of relapses and progression in neurological disability and confirmed a favorable safety profile by the low incidence of SAEs under real-world conditions in the Czech Republic.

## Data Availability

The data that support the findings of this study are available from [Novartis s.r.o.] but restrictions apply to the availability of these data, which were used under license for the current study, and so are not publicly available. Data are however available from the authors upon reasonable request and with permission of [Novartis s.r.o.].

## Abbreviations

AE: Adverse event
CI: Confidence interval
CNS: Central nervous system
EDSS: Expanded Disability Status Scale
KM: Kaplan–Meier
GOLEMS: Gilenya (FingOLimod) in prescribing conditions defined by the CzEch regulator of drug reiMburSement
S1P: Sphingosine 1-phosphate
SAE: Serious adverse event
WPAI-GH: Work Productivity and Activity Impairment Questionnaire-General Health
